# Prevalence of excess binaural broadband loudness summation in the hearing-impaired population and implications for hearing aid gain targets

**DOI:** 10.1371/journal.pone.0330517

**Published:** 2025-08-29

**Authors:** Florian Denk, Dirk Oetting, Matthias Latzel, Harald Bonsel, Hendrik Husstedt

**Affiliations:** 1 German Institute of Hearing Aids, Lübeck, Germany; 2 Hörzentrum Oldenburg, Germany; 3 Sonova, Stäfa, Switzerland; 4 Acousticon, Reinheim, Germany; LSU Health Shreveport, UNITED STATES OF AMERICA

## Abstract

Previous studies reported large individual differences in binaural broadband loudness summation in hearing-impaired listeners after narrowband loudness was normalized. These differences in loudness perception might require substantial fine-tuning for some hearing aid users to provide acceptable loudness in daily use. The present study aims at characterizing binaural broadband loudness summation for a hearing-impaired population, the prevalence of higher-than-normal values, and the potential implications for hearing aid target gains. For 180 hearing-impaired participants we measured standard audiological diagnostic parameters, binaural broadband loudness summation and computed gain targets according to NAL-NL2, DSLm[i/o] and trueLOUDNESS, a prescriptive procedure that includes individual loudness measurements. The observed binaural broadband loudness summation of the hearing-impaired participants was, on average, 13 dB higher and showed a higher variance relative to a normal-hearing reference group. In about 40% of all participants a binaural broadband loudness summation was beyond the normal-hearing range. The average excess loudness summation appears to be included in established prescriptive procedures, while relevant differences to trueLOUDNESS gain targets were observed in more than half of the participants. Elevated binaural broadband loudness summation appears to be a prevalent and individual trait in the hearing-impaired population and its assessment might be useful for individualized hearing aid fitting.

## 1. Introduction

Gain prescription procedures for hearing aids aim at compensating for perceptual effects of hearing impairment, including loss of audibility, impaired speech intelligibility and altered loudness perception, by application of frequency and level-dependent gain [1,2]. To obtain gain targets, most prescriptive procedures take the hearing threshold from the audiogram as the main input, sometimes supplemented by other metrics such as Uncomfortable Loudness Levels (UCL), air-bone-gaps or personal data like age or gender [3–5]. Although the potential benefits have been discussed in academic research for a considerable amount of time, no clinically used prescriptive procedure considers suprathreshold parameters other than UCLs. The current best-practice prescriptive procedures thus neglect the commonly accepted understanding that a lot of individual variation of audiological outcomes across hearing-impaired listeners exists that is not captured by the audiogram [6–10].

Common prescription procedures follow different approaches to compensating for the consequences of hearing loss [1]. NAL-NL2 aims at maximizing speech intelligibility while limiting the overall loudness to normal or less-than-normal [4,11]. DSLm[i/o] aims at loudness normalization based on the hearing threshold [5]. CAMEQ and CAM2 attempt to equalize the loudness of speech across frequency while limiting the overall loudness to a normal-hearing level [12]. All prescription procedures rely on computational models of the (impaired) auditory system for obtaining prescribed insertion gains. Such models are usually optimized to represent the average listener with a given audiogram, and in principle cannot account for individual supra-threshold factors. Therefore, the target gains computed by prescriptive procedures provide only initial fittings. In many cases, substantial fine-tuning of gains is necessary to reach a satisfactory outcome for the individual hearing aid user, for which no or only little formal guidance exists [1,3,13].

In the context of loudness-based hearing aid fitting, Oetting et al. [14] reported individual differences in loudness perception of binaurally presented broadband sounds, which could not be predicted based on monaural, narrowband loudness functions or hearing thresholds. This effect, henceforth referred to excess binaural broadband loudness summation, had not been observed in previous studies but was reproduced in several follow-up studies and using various methods of loudness assessment [15–19]. It should be noted here that this binaural broadband loudness summation should not be equated with binaural loudness summation as assessed at a single level using narrowband signals, which was typically found to be lower in hearing-impaired relative to normal-hearing listeners [1]. By considering the individual binaural broadband loudness summation in a dynamic compression framework controlled by the interaural level difference and bandwidth of the input signals, Oetting et al. [20] were able to restore normal loudness perception for both monaural and binaural broadband sounds in a number of listeners with variable binaural broadband loudness summation. Assuming that most relevant everyday sounds are broadband and binaurally received, the individual excess loudness summation together with narrowband loudness functions can be used to obtain individual prescriptive hearing aid gain targets. The procedure can be considerably speeded up by estimation of narrowband loudness functions from the audiogram, without significant effects on the resulting gain targets [21]. Values of the loudness functions for the normal-hearing reference data and the frequency and hearing threshold-dependent loudness functions are given by Zimmer et al. [19]. The resulting prescriptive procedure, termed trueLOUDNESS, takes as input the air-conduction hearing thresholds of both ears and the categorical loudness function for a binaurally presented female speech-shaped noise that is amplified according to narrowband loudness normalization based on the hearing thresholds. The individual aided loudness function thus gives insights to the level-dependent aided loudness perception relative to normal-hearing listeners. This information is then used to adapt the gain targets based on the underlying hearing threshold-based narrowband loudness normalization. Evaluation studies of trueLOUDNESS showed more normal loudness perception and equivalent speech intelligibility outcomes compared to NAL-NL2 [16,22]. However, previous studies assessed only a limited number of participants and did not systematically assess the differences between resulting gain targets and those for established prescription procedures.

In the present investigation, we aimed to characterize the prevalence of excess binaural broadband loudness summation in the hearing-impaired population, and the impact on target gain prescriptions. To this end, a multi-centre study was conducted to recruit participants as close as possible from the regular pool of hearing-impaired persons seeking hearing aid treatment. 160 participants were recruited and tested at five hearing aid professionals’ facilities (HAPFs) across Germany. Additionally, 20 participants that regularly participated in hearing aid-related studies were recruited from a database maintained by the German Institute of Hearing Aids (Deutsches Hörgeräte Institut, DHI) Lübeck, and tested in its laboratories using the same hardware as in the HAPFs. Further, a normal-hearing reference group (N = 20) underwent identical measurements at DHI. Measurements included an extended set of standard audiologic diagnostic parameters (see Methods section), the Inventory on Hyperacusis Symptoms (IHS) questionnaire [23], and categorical loudness scaling that included simulated narrowband loudness compensation [21]. Gain targets were computed for all participants using trueLOUDNESS, NAL-NL2 and DSLm[i/o]. This rich database allowed an assessment of the prevalence of excess loudness summation in the target population, potential audiological predictors of this parameter, and deviations of gain targets between established prescriptive procedures and trueLOUDNESS.

## 2. Measurements

### 2.1. Procedure

The study protocol was kept as close as possible to the hearing assessment component of a regular hearing aid fitting appointment in a HAPF in Germany. It included gathering of personal data (age, gender) and existing hearing aid provision (type of hearing aids and coupling, years of hearing aid experience). Audiometric measurements included air-conduction thresholds for sine tones at 11 audiometric frequencies between 125 Hz and 8 kHz, bone conduction thresholds and UCLs at 0.5, 1, 2 and 4 kHz, for the left and right ears. UCLs were also measured for speech (Freiburg polysyllabic numbers material) for the left and right ear monaurally as well as with diotic presentation, all measured through headphones. The participants completed also the IHS questionnaire on signs of hyperacusis [23].

In a second step, categorical loudness functions were measured for three stimuli, as outlined by Suck et al. [21], which is a shortened version of the procedure employed by Oetting et al. [14]. The stimuli, presented by the audiometer, included simulated non-linear hearing aid amplification and thus can be interpreted as aided measurements. The gains were derived from the difference between narrowband monaural loudness functions estimated from the audiogram and the appropriate loudness functions obtained with normal-hearing listeners, leading to an approximate narrowband loudness normalization [19,21]. Loudness functions were measured adaptively [24] and sequentially for three stimuli: 1) a uniformly exciting noise (UEN, creating equal power in each critical band according to [25]) with 5 critical bands bandwidth (800–1300 Hz), 2) a 17 critical bands wide UEN (500–4000 Hz) and 3) the International Female speech-shaped Noise (IFnoise) obtained from the International Speech Test Signal [26]. For further evaluations, only the results for the IFnoise are considered since it has the most speech-like spectrum of the test signals.

All measurements were conducted using an ACAM5 audiometer (Acousticon, Reinheim, Germany) with firmware version 5.22.13Rev5123 and the trueLOUDNESS module installed, which implements the loudness scaling including simulated amplification using the extra-aural A2000 headphone (Acousticon, Reinheim, Germany). Audiometric thresholds were measured using headphones that varied between measurement sites. All measurements were conducted by trained HAPFs in appropriately treated acoustic test rooms.

### 2.2. Multi-centre design and participant recruitment

The study aimed at recruiting a broad range of hearing-impaired participants representing the typical cohort of individuals seeking hearing aid treatment. Inclusion criteria were at least 18 years of age, an audiometric hearing loss of at least 30 dB between 0.5 and 4 kHz in the better ear, and a difference of Pure Tone Average (PTA) thresholds across 0.5, 1, 2, and 4 kHz between ears of no more than 15 dB. Participants could either be “New” (no more than 3 months after initial fit) or “Experienced” (at least two years of continuous use) hearing aid users, with the intention of recruiting approximately equal numbers in the two categories. Exclusion criteria were the use of any hearing implants.

Most measurements were conducted at five HAPFs across Germany. The study protocol was issued by the DHI, who supervised data collection and performed all evaluations. The study was approved by the Ethics Committee of the University of Lübeck (vote no. 2024−108). Participants were recruited by employees of the HAPFs among their regular clients on a voluntary basis. They gave written informed consent to participate in the study before any data collection beyond their regular appointment was made. Participants were recruited between February 1^st^ and July 22^nd^ of 2024. All collaborators were instructed to ignore any prior audiological history of the clients, except for meeting inclusion criteria. The collaborating HAPFs were reimbursed based on the number of contributed datasets. They were free to choose whether their participants were reimbursed. Data were transmitted to DHI through a password-protected cloud storage system in a pseudonymized way under a HAPF-specific client ID. In this way, only the HAPF was able to link the study data to a person. Personal data and IHS responses were inserted in an Excel document, while the results of audiometry and loudness scaling could be exported automatically from the audiometer software. A total of 160 hearing-impaired individuals participated through the HAPFs, including 88 New users.

Identical measurements were performed in laboratories of the DHI for a cohort of 20 experienced hearing aid users that regularly participated in hearing studies. This group is henceforth referred to “Experimenters” in the following. In addition, 20 young normal-hearing participants (18–25 of age, hearing thresholds max. 15 dB HL at no more than 2 frequencies between 0.25 8 kHz for both ears) underwent the measurements for reference purposes. The results from the normal-hearing participants reproduced previous data obtained with different hardware very well [14]. The data from the current evaluation were used as a normal-hearing reference.

### 2.3. Gain target computation

Based on the audiometric measurements, third-octave target insertion gains for a speech signal according to common prescriptive procedures were calculated for all participants. Gains were computed for the NAL-NL2 [3,4], DSL multistage input-output (DSLm[i/o]) [5] and trueLOUDNESS [20,21] prescription procedures. NAL-NL2 is probably the most commonly used non-proprietary prescription rule in both research and clinical applications, and was developed to maximize speech intelligibility while keeping loudness normal or less-than-normal. NAL-NL2 gains were computed using its dll engine (Version 2.15) and the openMHA NAL-NL2 wrapper [27] to call the 32-Bit dll engine out of Matlab R2024b. All air- and bone-conduction thresholds and the participant’s age and gender were used as input. Target insertion gains were computed for a bilateral fitting, a non-tonal language, slow compression speed, and an experienced adult user. The rationale of the DSLm[i/o] gain prescription is to achieve audibility by transforming input levels to the reduced audible dynamic range while avoiding loudness discomfort. DSLm[i/o] gain targets were computed through an automated access to an online tool (https://core.dslio.com/), using the audiograms and UCLs as input, with no wideband correction and for the quiet program. DSLm[i/o] by default assumes different spectral shapes of the input signal at different input levels to account for effects of vocal effort. To be able to compare insertion gain targets to those for the other prescription procedures, we used IFnoise spectra at appropriate levels as input for DSLm[i/o]. Note that bone-conduction thresholds are not used by the DSLm[i/o] algorithm if UCL measurements are available.

The trueLOUDNESS prescription procedure aims at normalizing a categorical loudness function for broadband binaural sounds, with gain targets computed in a three-step process. First, initial gains are derived from the difference between monaural narrowband normal-hearing loudness functions and appropriate loudness functions estimated from the audiogram, and represent narrowband loudness normalization [21]. Second, a loudness function for a broadband, binaural stimulus including these initial gains is measured as described above. Third, the gains are adapted based on this loudness function, as described by Oetting et al. [20], here using a full bandwidth and no binaural difference. Gain targets according to trueLOUDNESS were computed on the ACAM5 audiometer and exported together with the loudness functions.

Target gains were computed for input levels of 50, 65, and 80 dB SPL broadband speech for all frequencies returned by the utilized prescriptive procedures. Comparisons were made for insertion gains at 500, 1000, 2000 and 4000 Hz. It was a-priori determined that a meaningful difference between gain targets was deemed to occur if the unsigned difference between prescription procedures, averaged across all considered levels, frequencies and both ears, exceeded 5 dB. The compression ratio applied between a *high* and *low* broadband input level $L_{in}$ at each frequency was computed based on the gains G for these input levels


$$CR_{high-low\;}\;=\frac{\Delta L_{in}}{\Delta L_{in}+G\left(L_{in}^{\left(high\right)}\right)-G\left(L_{in}^{\left(low\right)}\right)}.$$ (1)

## 3. Results & analysis

### 3.1. Characteristics of participant population

**Fig 1:**
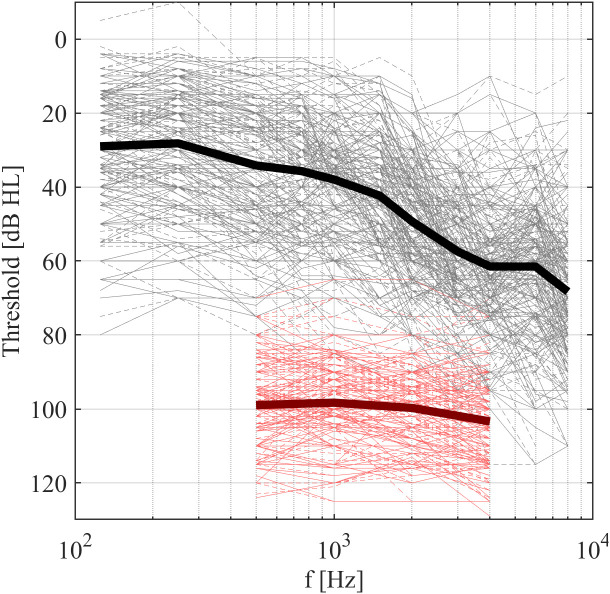
Air-conduction thresholds (black) and UCLs (red) for all participants, thick lines showing averages. Solid lines show data for left ears, dashed lines for right ears.

Fig 1 shows the pooled air conduction hearing thresholds and UCLs of all participants. The thresholds covered a wide range from moderate to profound hearing loss, with an average close to the N3 type hearing loss [28]. The UCLs varied between 80 and 120 dB HL, with an average close to 100 dB HL across all frequencies used.

**Fig 2:**
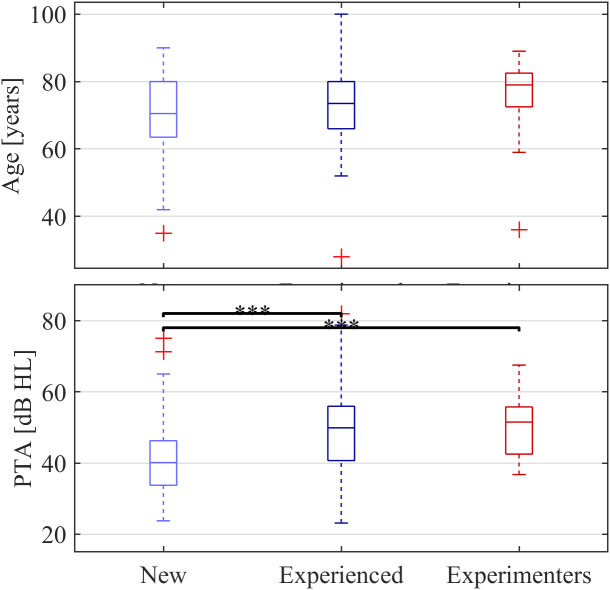
Distribution of age and PTA thresholds for each participant group. Horizontal lines indicate the median, boxes the interquartile range, and whiskers the full data range excluding outliers (crosses). Brackets with asteriscs above denote significant differences (*p* < 0.001).

Fig 2 shows the age and PTA hearing thresholds distribution of the participants for each predefined group. Differences between groups were assessed using unpaired *t*-tests with Bonferroni corrections. The PTA differed significantly between New users and the other two groups (both *p* < 0.001), the New users having better PTA thresholds. There was no significant difference between the Experienced users and the Experimenters group. There was no significant difference in age between the groups. However, there is a trend that the New users were the youngest (70.9 ± 11.4 years, mean + standard deviation), followed by the Experienced users from the field (72.7 ± 11.3 years), and the Experimenters group, which had the highest age (75.9 ± 12.3 years).

### 3.2. Excess binaural broadband loudness summation

**Fig 3:**
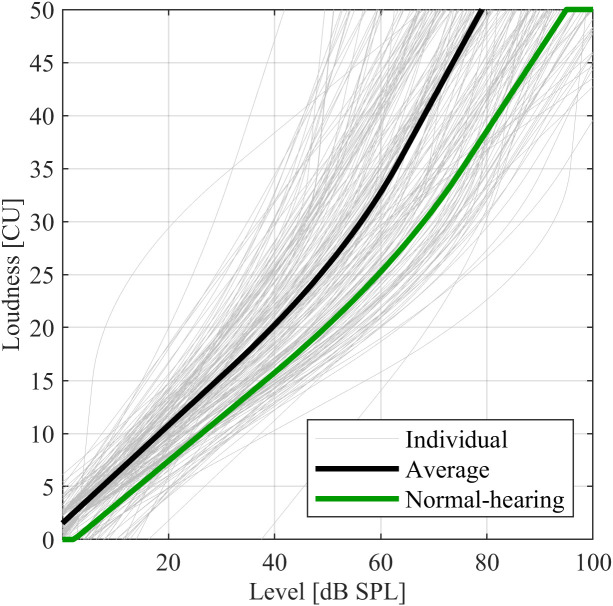
Loudness functions for IFnoise with narrowband loudness normalization for hearing-impaired participants (gray lines, thick black line showing average) and normal-hearing reference function (thick green line).

Fig 3 shows categorical loudness functions for the hearing-impaired participants for the IFnoise signal, the average (level averaged for each Categorical Unit, CU), and the normal-hearing reference function. There was a wide distribution of loudness functions, the variability increasing with input level. 95% of the levels for a rating of 40 CU (between “loud” and “very loud”) after simulated amplification fell between 45.8 and 94.3 dB SPL, with an average of 69.3 dB SPL. The corresponding values for normal-hearing listeners were 63.6 to 102.8 dB SPL, with an average of 82.3 dB SPL.

For further evaluations, the difference in input level at 40 CU ($\Delta L_{40})$ between a given participant ($L_{40})$ and the normal-hearing average ($L_{40}^{ref};$ is considered


$$\Delta L_{40}=L_{40}^{ref}-L_{40}.$$ (2)

**Fig 4:**
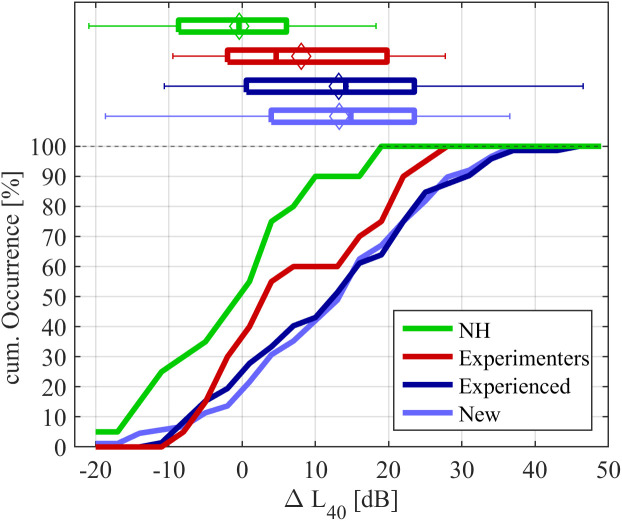
Distribution of binaural broadband loudness summation, showing cumulative plots (bottom part) and boxplots (top part) for each group. Diamonds in the boxplots denote the mean of the distributions.

A positive value of $\Delta L_{40}$ indicates that a rating of 40 CU was reached at lower input levels than the average for the normal-hearing group, representing a loudness for binaural broadband stimuli that is higher than expected from the hearing threshold-based narrowband loudness functions. $\Delta L_{40}$ can be interpreted as a metric for the individual binaural broadband loudness summation. Fig 4 shows the cumulative distributions and boxplots of $\Delta L_{40}$ for each group. The distribution of normal-hearing participants is centred around the average defined as 0 dB, with extreme cases deviating by about 15 dB. For all hearing-impaired participant groups, the distributions were shifted to the right, indicating increased binaural broadband loudness summation. The distributions for New and Experienced participants recruited in HAPFs were similar but differed notably from that of the Experimenters recruited form the research database. For the participants from the HAPFs, the distribution of $\Delta L_{40}$ was shifted to the right, indicating a higher proportion of individuals with high binaural broadband loudness summation. The average values for $\Delta L_{40}$ were 8.1 dB for the Experimenters, and 13.2 dB for both the New and Experienced participants. Unpaired *t*-tests did not show significant differences between the predefined hearing-impaired participant groups, in spite of these visible trends (*p* > 0.1).

Among the hearing-impaired participants, the values of $\Delta L_{40}$ fell between –12.4 and 36.1 dB (2.5 and 97.5 percentiles), showing the result of the majority of hearing-impaired participants fell within the normal-hearing range. However, the 95^th^ percentile of the normal-hearing data ($\Delta L_{40}=$ 17.2 dB) as a boundary value, higher-than-normal binaural broadband loudness summation occurred for 30% of the Experimenters, and 38 and 39% of New and Experienced hearing aid users, respectively.

**Fig 5:**
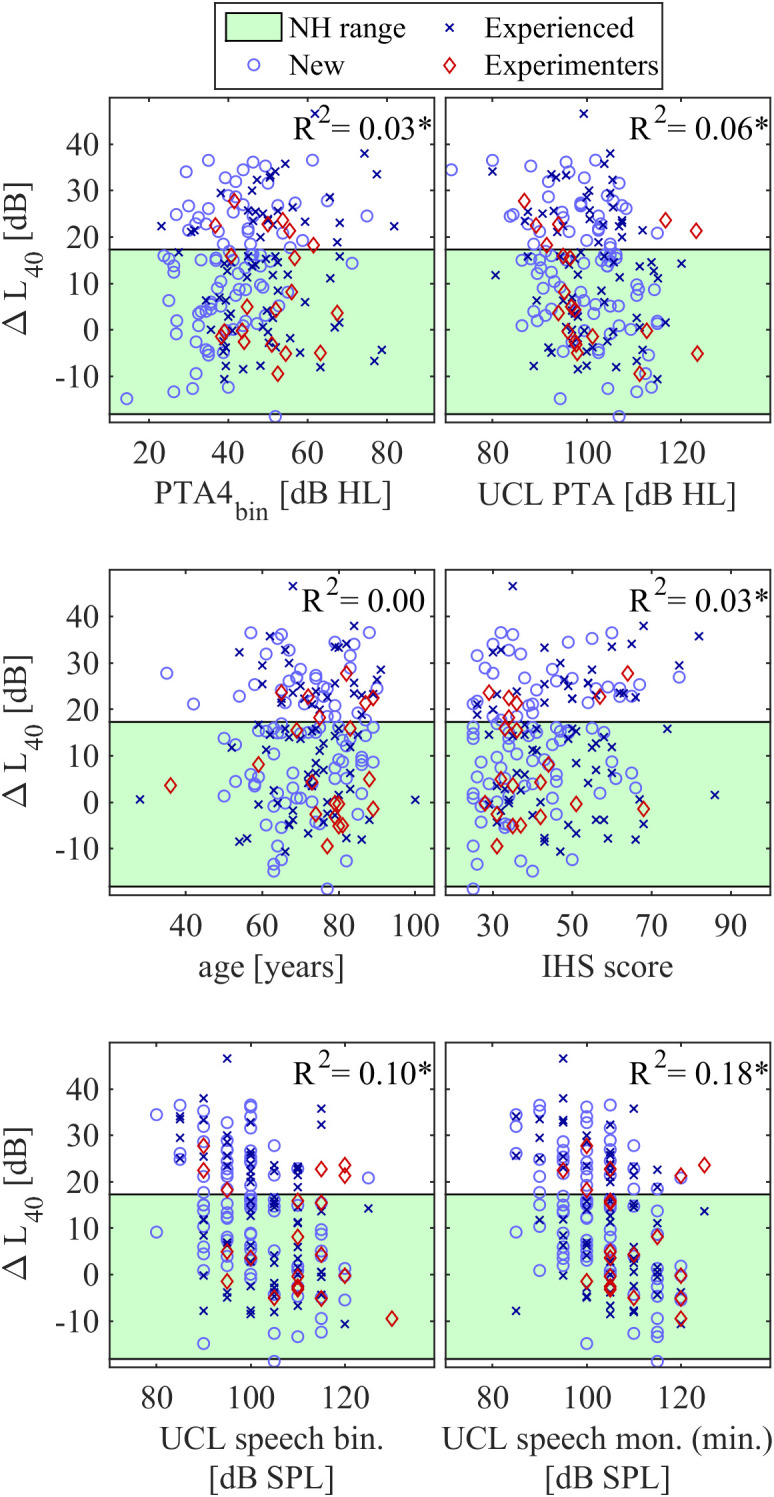
Correlations between the individual excess binaural broadband loudness summation $\Delta L_{40}$ and other obtained audiological parameters (see x-axes). Green shaded area denotes the 90% range for normal-hearing participants. Text in the top right corners shows coefficients of determination, stars indicate significant correlations.

Fig 5 shows scatter plots between $\Delta L_{40}$ and other audiological parameters. The correlations were significant for the binaurally averaged PTA hearing threshold ($R^{2}=\mathrm{.03},\;p=\mathrm{.033})$, the IHS score ($R^{2}=\mathrm{.03},\;p=\mathrm{.030})$, the binaurally averaged PTA UCL ($R^{2}=\mathrm{.06},\;p<\mathrm{.001})$, the UCL for the speech signal presented binaurally ($R^{2}=\mathrm{.10},p<\mathrm{.001})$, and the minimum of the speech signal UCL across ears with monaural presentation $\left(R^{2}=\mathrm{.18},\;p<\mathrm{.001}\right)$. Although the correlations were significant, the coefficients of determination were too low to indicate any predictive value on an individual basis. There was no significant correlation between age and $\Delta L_{40}$. $\Delta L_{40}$ scores were well-distributed across the observed scale also in participants with likely hyperacusis, considering either the original threshold of 69 [23] or the more liberal 56 suggested by Azah et al., [29] (Fig 5, middle right panel). A multi-linear regression model was fitted to the data and gave an adjusted coefficient of determination of $R^{2}=0.21$. Thus, no linear combination of the predictors resulted in a significantly better prediction than the minimum monaural speech UCL.

### 3.3. Prescribed target gain

**Fig 6:**
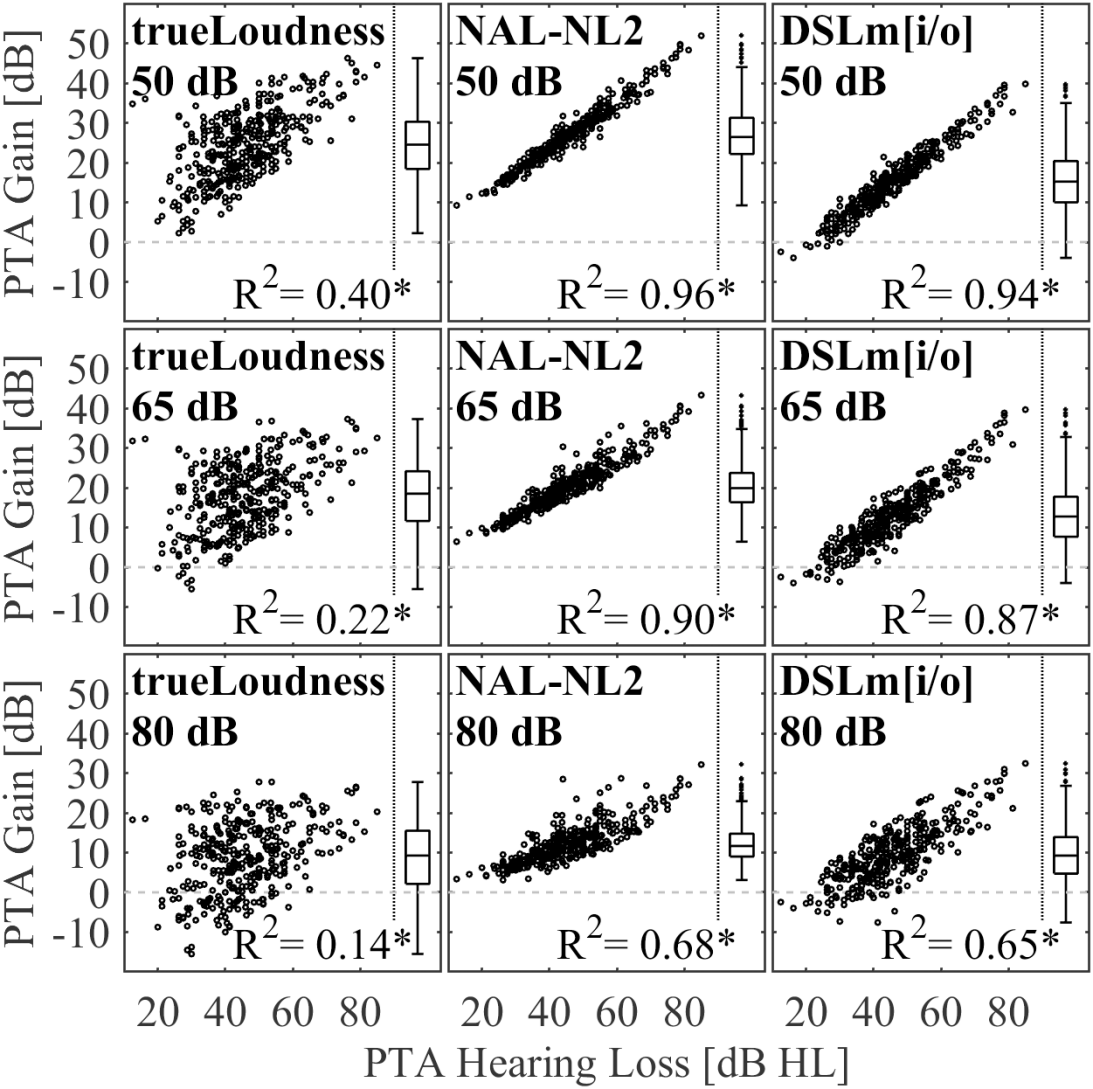
Scatter plots of PTA gains against PTA hearing loss for each prescription rule and input level with given $R^{2}$ values. Boxplots show the distribution of PTA gains.

Fig 6 shows scatter plots of the PTA hearing loss against average target gain at the PTA frequencies 0.5, 1, 2, and 4 kHz for each prescription procedure and input level, together with boxplots of the average target gain distributions. Each point represents one ear. For NAL-NL2 and DSLm[i/o], the correlations were high for input levels of 50 and 65 dB SPL, reaching coefficients of determination around and above 0.9. This shows that these gains were prescribed mostly based on the threshold. For the input level of 80 dB SPL, the coefficients of determination reduced to $R^{2}=0.6$ and $R^{2}=0.68$ for DSLm[i/o] and NAL-NL2, respectively, showing a significant influence of other parameters. For DSLm[i/o], the parameter is probably the UCL, while in NAL-NL2 the air-bone gap is used to complement the threshold to estimate the UCL. For trueLOUDNESS, the coefficients of determination between target gains and thresholds were generally lower, being $R^{2}=0.4$ at 50 dB SPL input, and declining to $R^{2}=0.14$ at 80 dB SPL. Although these correlations were statistically significant, this shows that the hearing threshold has only minor influence on the trueLOUDNESS target gains for input levels of 65 and 80 dB SPL.

It should be noted that the range of gain values across all participants was similar for all prescription procedures for the input levels of 50 and 65 dB SPL. However, for a given PTA hearing losses the range was much higher for trueLOUDNESS than for NAL-NL2 or DSLm[i/o]. At an input level of 80 dB SPL, the range of gains was smaller for NAL-NL2 than for the other two gain rules, which prescribed similar maximum gains but lower minimum gains. Other than NAL-NL2, both trueLOUDNESS and DSLm[i/o] prescribed negative gain targets for some individuals, most often for the input level of 80 dB SPL and for PTA hearing thresholds less than 50 dB HL.

**Fig 7:**
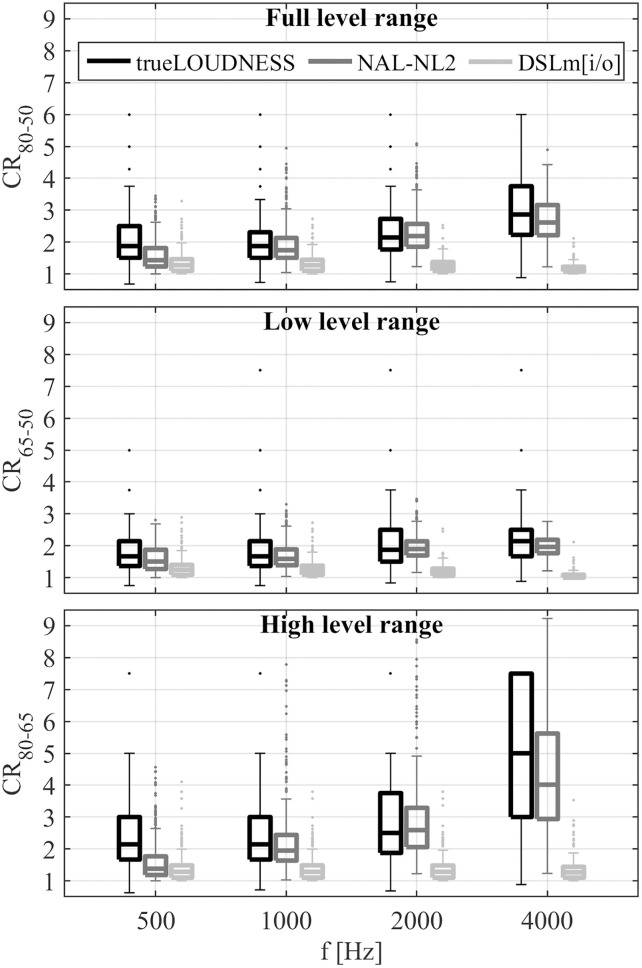
Boxplots of compression ratios at each frequencies across several level ranges in the regarded gain as specified in eq. (1) for level range given in the panel title and y-label. Boxplots show distributions of compression ratios from gain targets for the given frequency determined in all ears of New and Experienced participants (320 ears).

Fig 7 shows boxplots of compression ratios across ears for several frequencies and level ranges for each regarded gain rule. Across all gain rules and frequencies, the compression ratios were smaller for the low- and full- level range than for the high-level range. The compression ratios for DSLm[i/o] were generally smaller than those for NAL-NL2 and trueLOUDNESS, and exceeded 2 only for a few ears. For trueLOUDNESS and NAL-NL2, compression ratios tended to increase with frequency up to 4 kHz, corresponding to the average hearing thresholds that increased with frequency (Fig 1). The compression ratios for trueLOUDNESS were, on average, similar to those for NAL-NL2, but tended to be higher at 500 Hz (and frequencies below, not shown in Fig 7) and have a higher upper quartile range. Also, trueLOUDNESS showed cases with the lowest compression ratios among all gain rules, with compression ratios below 1. The highest median compression ratios, between 4–5 with both NAL-NL2 and trueLOUDNESS, occurred for the high-level range at 4 kHz, with many cases exceeding a target compression ratio of 7. It should be noted that many current commercial hearing aids prohibit the use of compression ratios in this range.

**Fig 8:**
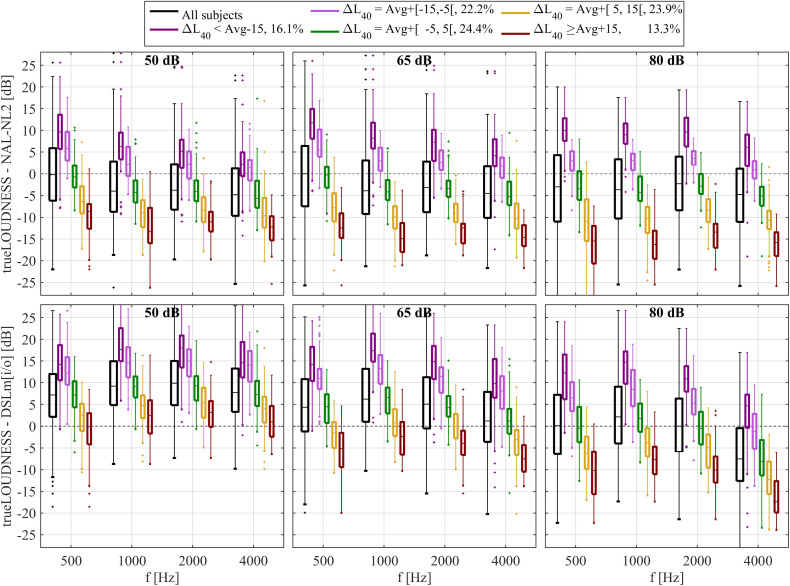
Boxplots of the gain difference between trueLOUDNESS and NAL-NL2 (top row), and between trueLOUDNESS and DSLm[i/o] (bottom row) for each input level (see panel title) and frequency. Black boxplots show distributions across all ears, colors indicate grouping by the excess binuaral broadband loudness summation (see legend). Percentages in the legend denote the percentage of participants falling into each category.

Fig 8 shows boxplots of the differences in target gains between trueLOUDNESS and the other two rules, for each input level and frequency. The differences are shown both across all participants (black), and grouped by the individual values of $\Delta L_{40}$. Across participants, the median differences between trueLOUDNESS and NAL-NL2 were below 5 dB, with a general tendency that trueLOUDNESS prescribed lower gains than NAL-NL2, except for 500 Hz (and below, not shown here) at low and intermediate input levels. Differences between these two prescriptive procedures for individual ears reached 20 dB in either direction, for all input levels and frequencies. The individual differences are explained largely by $\Delta L_{40}$. TrueLOUDNESS gains for individuals with an average or slightly below average $\Delta L_{40}$ (green and light purple boxes in Fig 8) were close to those for NAL-NL2, while a high $\Delta L_{40}$ values led to trueLOUDNESS gains lower than those prescribed by NAL-NL2 and vice versa.

The differences between DSLm[i/o] and trueLOUDNESS gains depended on the input level and frequency. In the median, the 50 dB SPL input level, trueLOUDNESS gains were 7–10 dB higher than DSLm[i/o] gains. This difference declined at higher input levels and was close to 0 dB for the 80 dB SPL input level, except at 4 kHz, where DSLm[i/o] prescribed a 5 dB lower median gain. Individual deviations spanned a range close to 50 dB, showing similar dependence on $\Delta L_{40}$ as NAL-NL2. Based on correlation analysis of gains averaged across levels and frequencies, 76% and 88% of the difference between trueLOUDNESS and DSL and NAL-NL2 can be explained by $\Delta L_{40}$, respectively.

**Fig 9:**
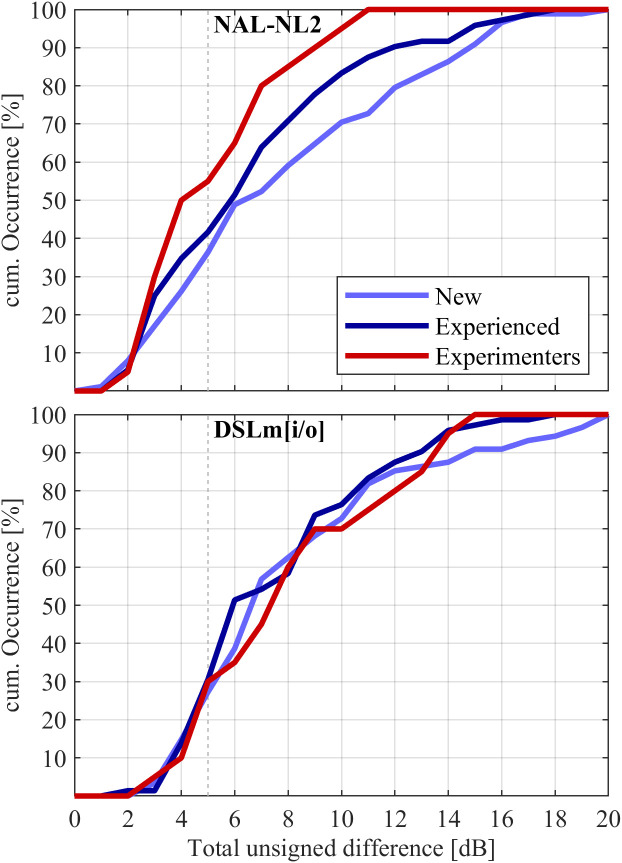
Cumulative occurrence of gain differences between trueLOUDNESS and other fitting rules as denoted by panel title based on the unsigned average of differences across 500, 1000, 2000 and 4000 Hz, input levels of 50, 65 and 80 dB, and both ears of a given participant.

Fig 9 shows cumulative distributions of the average unsigned difference (see 2.3) between trueLOUDNESS and the two other prescriptive procedures. The predefined significant difference of 5 dB was exceeded for NAL-NL2 by about 45% of Experimenters participants, and 60% of both New and Experienced participants from the HAPFs. The difference between trueLOUDNESS and NAL-NL2 tended to be largest for the New participants (the shallowest curve), followed by Experienced listeners and the Experimenters. Differences exceeding 10 dB occurred rarely for the Experimenters, but occurred for about 18% of the Experienced and 30% of the New participants. For the DSLm[i/o] rule, there was no obvious difference between participant groups, and the 5 dB difference was exceeded for about 65%. Large differences exceeding 10 dB occurred for about 28% of all participants.

## 4. Discussion

### 4.1. Prevalence of increased binaural broadband loudness summation in hearing-impaired listeners

A non-negligible proportion of hearing-impaired individuals showed binaural broadband loudness summation exceeding the range of normal-hearing listeners. That is, using categorical loudness scaling with a binaural broadband sound and level-dependent amplification aiming at narrowband loudness normalization based on the individual hearing thresholds, the sensitivity for loud sounds (at 40 CU) was about 13 dB greater for the average hearing-impaired individual than for the normal-hearing average. This difference decreased for softer sounds and increased for higher input levels. There were large variations among hearing-impaired people, with values ranging from 10 dB below the normal-hearing average to 40 dB above it. Binaural broadband loudness summation can be regarded as above the normal-hearing range for 30–40% of hearing-impaired individuals. We conclude that excess binaural broadband loudness summation is a common and highly individual trait among hearing-impaired listeners.

Excess binaural broadband loudness summation tended to be lower for the Experimenters group recruited from a database of people who regularly participated in hearing studies than for those recruited in the HAPFs. While this difference failed to reach statistical significance, we regard this result as relevant, given the visible differences of distributions. The distribution of excess binaural broadband loudness summation for the Experimenters group was in excellent agreement with previous assessments of this effect (see supplemental S1 Fig) conducted with participants from an independent but similar database of hearing-impaired who regularly participated in hearing studies [20,30]. We speculate that the lower excess binaural broadband loudness summation in this group of participants might be caused by a sampling bias. It could be explained by the fact that hearing aid users with high excess binaural broadband loudness summation, who might perceive many situations as too loud, would be less inclined to regularly participate in hearing-aid-related listening experiments. Potential sampling biases as noted here should be considered in further studies employing hearing-impaired listeners. The present result emphasizes the need to recruit participants as close as possible to clinical practice in order to obtain representative results.

Best efforts were made to recruit typical patients seeking hearing aid treatment by inviting them to participate during a hearing aid fitting session, with only minor extra effort and time for them. However, sampling biases cannot be ruled out for these groups of participants either. First, the data were obtained at the rather few HAPFs equipped to perform loudness scaling. They were members of a group of the first hearing aid professionals that regularly used the trueLOUDNESS prescription procedure, and it can be assumed they were personally convinced of this approach. It can thus not be ruled out that the participants were influenced unintentionally by the operator of the experiment, although we consider the likelihood rather low given that the scaling measurement was highly automated. Although the participating hearing aid professionals were explicitly instructed to invite participants regardless of their audiological outcomes, a bias towards recruiting participants with excess binaural broadband loudness summation cannot be ruled out completely. One observation that speaks against such a bias is that New and Experienced participants showed similar distributions of excess binaural broadband loudness summation, and the characteristics of the New participants were unknown before they were recruited for the experiments. It should be noted that some degree of loss of control about the experimental parameters is inevitably connected to data collection in the field as conducted in the present study. The present results highlight the importance of including listeners that would not be willing or be able to (regularly) participate in laboratory studies in experiments characterizing hearing-impaired individuals.

We found no audiological diagnostic parameter that predicted the excess binaural broadband loudness summation by itself or in a linear combination sufficiently accurate to be clinically relevant (see Fig 5). Surprisingly, this also included also the IHS questionnaire on hyperacusis [23], whose scores were expected to be correlated with excess loudness especially for loud environments. We interpret this as indication that hyperacusis is a condition where loud sounds generally lead to high discomfort, which should not be equated with elevated loudness perception when using hearing aids. Also, it may seem surprising that the correlation between the binaural broadband loudness summation measured by $\Delta L_{40}$ was only weakly correlated with UCLs measured with broadband speech signals (see Fig 5). Two factors may have contributed to the low correlation. First, variations in threshold were not compensated in the UCL measurement with speech, leading to reduced spectral loudness summation [14]. A different measurement signal such as pink noise could lead to more uniform simultaneous excitation of multiple auditory bands and induce higher binaural broadband loudness summation than speech. Second, the UCL is based on a subjective response that shows significant variance [31], with a procedure deviating from a loudness scaling measurement. While a UCL measurement with an appropriate broadband test signal could be faster and easier to implement than loudness scaling at different frequencies, future research is required to determine the potential use of broadband binaural UCLs as a fast means to characterize individual binaural broadband loudness summation.

### 4.2. Implications for hearing aid gain targets

The present data show clearly that individual gain targets obtained using trueLOUDNESS may substantially deviate from established prescriptive procedures. This deviation exceeded a predefined significance criterion for approximately half of all participants. While the average of the trueLOUDNESS gain targets was close to that for NAL-NL2, systematic differences between trueLOUDNESS and DSLm[i/o] were observed. Compared to trueLOUDNESS, DSLm[i/o] prescribed significantly lower gains for the 50 and 65 dB SPL inputs, leading to compression ratios that were generally lower in DSLm[i/o]. The gain targets between trueLOUDNESS and NAL-NL2 were most consistent for participants with a $\Delta L_{40}$ in the range of 15 dB below to 5 dB above the hearing-impaired average of 13 dB. A correlation analysis showed that the minimum difference between trueLOUDNESS and NAL-NL2 occurred for listeners with $\Delta L_{40}$ around 10 dB. Thus, it could be stated that NAL-NL2 already includes average effects of excess binaural broadband loudness summation in hearing-impaired participants, providing further evidence for the existence of excess binaural broadband loudness summation in hearing-impaired listeners compared to the normal-hearing population. It should also be noted that the range of ±15 dB individual fine-tuning adjustments to NAL-NL2 reported by Keidser et al. [3] to achieve comfortable loudness matches well the range of differences between NAL-NL2 and trueLOUDNESS in the present investigation, while such gain adjustments are typically not applied within clinical fine tuning. The highly individual difference between prescription procedures is a direct consequence of additional diagnostic information independent of the threshold that influences the trueLOUDNESS target, while NAL-NL2 and DSLm[i/o] mainly rely on the threshold.

The present results show that trueLOUDNESS leads to a higher degree of individualization in the target gains. However, this study did not assess whether this higher degree of individualization leads to improved audiological outcomes and/or subjective preference. However, the observed differences between gain rules can be expected to result in clearly distinguishable settings for most participants, especially those with a binaural broadband loudness summation that deviates from the hearing-impaired average. Future research is needed to evaluate whether the trueLOUDNESS prescription procedure indeed provides better individualization of gain targets to the specific listener, which should be measurable as improved loudness perception in everyday environments, higher subjective preference and better or equivalent speech intelligibility outcomes.

## 5. Conclusions

The present investigation showed that excess binaural broadband loudness summation, relative to the normal-hearing population, is a prevalent but highly individual trait in hearing-impaired listeners. About 40% of hearing-impaired individuals showed loudness summation beyond the normal-hearing range, with an average of 13 dB input level difference for a loud sound, and individual values up to 35 dB. There was a trend indicating the distributions of this excess loudness summation varied between populations of hearing-impaired participants recruited in HAPFs and in a research database, showing the importance of recruitment strategies that should be considered in further studies more generally. A prescriptive procedure taking individual broadband binaural loudness summation into account led to large individual deviations from standard prescription procedures that would be meaningful for more than half of all participants, while average gain targets across participants were reasonably similar.

## Supporting information

S1 FigAbove shows the same information as Fig 4 from the main manuscript, but additionally includes the cumulative distribution of $\Delta L_{40}$ for a dataset of 324 hearing-impaired participants measured at the Hörzentrum Oldenburg (HZOL) during the development of the trueLOUDNESS procedure.All participants were part of a database of hearing-impaired individuals maintained by the HZOL, which were regularly invited to participate in hearing experiments. The distribution is in good agreement with the Experimenters group in the present dataset, which comprised 20 subjects from a similar database maintained by the German Institute of Hearing Aids, Lübeck.(PDF)

## References

[1] Dillon H. Hearing aids. 2 ed. Turramurra: Boomerang Press. 2012.

[2] Kollmeier B, Kiessling J. Functionality of hearing aids: state-of-the-art and future model-based solutions. Int J Audiol. 2018;57(sup3):S3–28. doi: 10.1080/14992027.2016.1256504 27951738

[3] Keidser G, Dillon H, Carter L, O’Brien A. NAL-NL2 empirical adjustments. Trends Amplif. 2012;16(4):211–23. doi: 10.1177/1084713812468511 23203416 PMC4040825

[4] Keidser G, Dillon H, Flax M, Ching T, Brewer S. The NAL-NL2 Prescription Procedure. Audiol Res. 2011;1(1):e24. doi: 10.4081/audiores.2011.e24 26557309 PMC4627149

[5] Scollie S, Seewald R, Cornelisse L, Moodie S, Bagatto M, Laurnagaray D, et al. The Desired Sensation Level multistage input/output algorithm. Trends Amplif. 2005;9(4):159–97. doi: 10.1177/108471380500900403 16424945 PMC4111494

[6] Jepsen ML, Dau T. Characterizing auditory processing and perception in individual listeners with sensorineural hearing loss. J Acoust Soc Am. 2011;129(1):262–81. doi: 10.1121/1.3518768 21303008

[7] Kiessling J, Schubert M, Archut A. Adaptive fitting of hearing instruments by category loudness scaling (ScalAdapt). Scand Audiol. 1996;25(3):153–60. doi: 10.3109/01050399609047998 8881002

[8] Kortlang S, Mauermann M, Ewert SD. Suprathreshold auditory processing deficits in noise: Effects of hearing loss and age. Hear Res. 2016;331:27–40. doi: 10.1016/j.heares.2015.10.004 26471199

[9] Saak S, Huelsmeier D, Kollmeier B, Buhl M. A flexible data-driven audiological patient stratification method for deriving auditory profiles. Front Neurol. 2022;13:959582. doi: 10.3389/fneur.2022.959582 36188360 PMC9520582

[10] Sanchez Lopez R, Bianchi F, Fereczkowski M, Santurette S, Dau T. Data-Driven Approach for Auditory Profiling and Characterization of Individual Hearing Loss. Trends Hear. 2018;22:2331216518807400. doi: 10.1177/2331216518807400 30384803 PMC6236853

[11] Byrne D, Dillon H, Ching T, Katsch R, Keidser G. NAL-NL1 Procedure for Fitting Nonlinear Hearing Aids: Characteristics and Comparisons with Other Procedures. J Am Acad Audiol. 2001;12(01):37–51. doi: 10.1055/s-0041-1741117 11214977

[12] Moore BCJ, Glasberg BR, Stone MA. Development of a new method for deriving initial fittings for hearing aids with multi-channel compression: CAMEQ2-HF. Int J Audiol. 2010;49(3):216–27. doi: 10.3109/14992020903296746 20151930

[13] Anderson MC, Arehart KH, Souza PE. Survey of Current Practice in the Fitting and Fine-Tuning of Common Signal-Processing Features in Hearing Aids for Adults. J Am Acad Audiol. 2018;29(2):118–24. doi: 10.3766/jaaa.16107 29401059 PMC6366669

[14] Oetting D, Hohmann V, Appell J-E, Kollmeier B, Ewert SD. Spectral and binaural loudness summation for hearing-impaired listeners. Hear Res. 2016;335:179–92. doi: 10.1016/j.heares.2016.03.010 27006003

[15] Ewert SD, Oetting D. Loudness summation of equal loud narrowband signals in normal-hearing and hearing-impaired listeners. Int J Audiol. 2018;57(sup3):S71–80. doi: 10.1080/14992027.2017.1380848 28971746

[16] Oetting D, Bach J-H, Krueger M, Vormann M, Schulte M, Meis M. Subjective loudness ratings of vehicle noise with the hearing aid fitting methods NAL-NL2 and trueLOUDNESS. Proceedings of the International Symposium on Auditory and Audiological Research. Nyborg, Denmark; 2019. pp. 289–96. Available: https://proceedings.isaar.eu/index.php/isaarproc/article/view/2019-33

[17] van Beurden M, Boymans M, van Geleuken M, Oetting D, Kollmeier B, Dreschler WA. Uni- and bilateral spectral loudness summation and binaural loudness summation with loudness matching and categorical loudness scaling. Int J Audiol. 2021;60(5):350–8. doi: 10.1080/14992027.2020.1832263 33100070

[18] van Beurden M, Boymans M, van Geleuken M, Oetting D, Kollmeier B, Dreschler WA. Potential Consequences of Spectral and Binaural Loudness Summation for Bilateral Hearing Aid Fitting. Trends Hear. 2018;22:2331216518805690. doi: 10.1177/2331216518805690 30353784 PMC6201175

[19] Zimmer J, Hartog L, Oetting D, Pöntynen H, Dietz M. Loudness and lateralization of binaural broadband noise for subjects with asymmetric hearing loss. GMS Z Audiol. 2024;6:Doc10. doi: 10.3205/zaud000045

[20] Oetting D, Hohmann V, Appell J-E, Kollmeier B, Ewert SD. Restoring Perceived Loudness for Listeners With Hearing Loss. Ear Hear. 2018;39(4):664–78. doi: 10.1097/AUD.0000000000000521 29210810

[21] Suck LC, Hartog L, Ewert SD, Hohmann V, Oetting D. Verkürzung der trueLOUDNESS Anpassmethode zur binauralen breitbandigen Lautheitsnormalisierung in Hörgeräten. 23 Jahrestagung der Deutschen Gesellschaft für Audiologie. Köln; 2020.

[22] Kramer F, Oetting D, Schädler MR, Hohmann V, Warzybok A. Speech recognition and loudness perception in normal-hearing and hearing-impaired listeners. In: Forum Acusticum, 2020. 3493. https://hal.science/hal-03235936/

[23] Greenberg B, Carlos M. Psychometric Properties and Factor Structure of a New Scale to Measure Hyperacusis: Introducing the Inventory of Hyperacusis Symptoms. Ear Hear. 2018;39(5):1025–34. doi: 10.1097/AUD.0000000000000583 29742543

[24] Brand T, Hohmann V. An adaptive procedure for categorical loudness scaling. J Acoust Soc Am. 2002;112(4):1597–604. doi: 10.1121/1.1502902 12398465

[25] Zwicker E. Subdivision of the Audible Frequency Range into Critical Bands (Frequenzgruppen). The Journal of the Acoustical Society of America. 1961;33(2):248–248. doi: 10.1121/1.1908630

[26] Holube I, Fredelake S, Vlaming M, Kollmeier B. Development and analysis of an International Speech Test Signal (ISTS). Int J Audiol. 2010;49(12):891–903. doi: 10.3109/14992027.2010.506889 21070124

[27] Herzke T. openMHA NAL-NL2 Wrapper. GitHub. https://github.com/HoerTech-gGmbH/openMHA/blob/master/README_NALNL2.md. 2023.

[28] Bisgaard N, Vlaming MSMG, Dahlquist M. Standard audiograms for the IEC 60118-15 measurement procedure. Trends Amplif. 2010;14(2):113–20. doi: 10.1177/1084713810379609 20724358 PMC4111352

[29] Aazh H, Danesh AA, Moore BCJ. Internal Consistency and Convergent Validity of the Inventory of Hyperacusis Symptoms. Ear Hear. 2021;42(4):917–26. doi: 10.1097/AUD.0000000000000982 33259445

[30] Oetting D. Die binaurale breitbandige Lautheitssummation. Hörakustik. 2021;09:8–13.

[31] Punch J, Joseph-A, Rakerd B. Most comfortable and uncomfortable loudness levels: six decades of research. Am J Audiol. 2004;13(2):144–57. doi: 10.1044/1059-0889(2004/019 15903140

