# Supplementary material for: Prevalence of excess binaural broadband loudness summation in the hearing-impaired population and implications for hearing aid gain targets
Source: PLoS One. 2025 Aug 29;20(8):e0330517. doi: 10.1371/journal.pone.0330517 (PMC12396666; doi:10.1371/journal.pone.0330517)
Supplement: S1 Fig — All participants were part of a database of hearing-impaired individuals maintained by the HZOL, which were regularly invited to participate in hearing experiments. The distribution is in good agreement with the Experimenters group in the present dataset, which comprised 20 subjects from a similar database maintained by the German Institute of Hearing Aids, Lübeck. (PDF) [file pone.0330517.s001.pdf]

Supplemental Plot

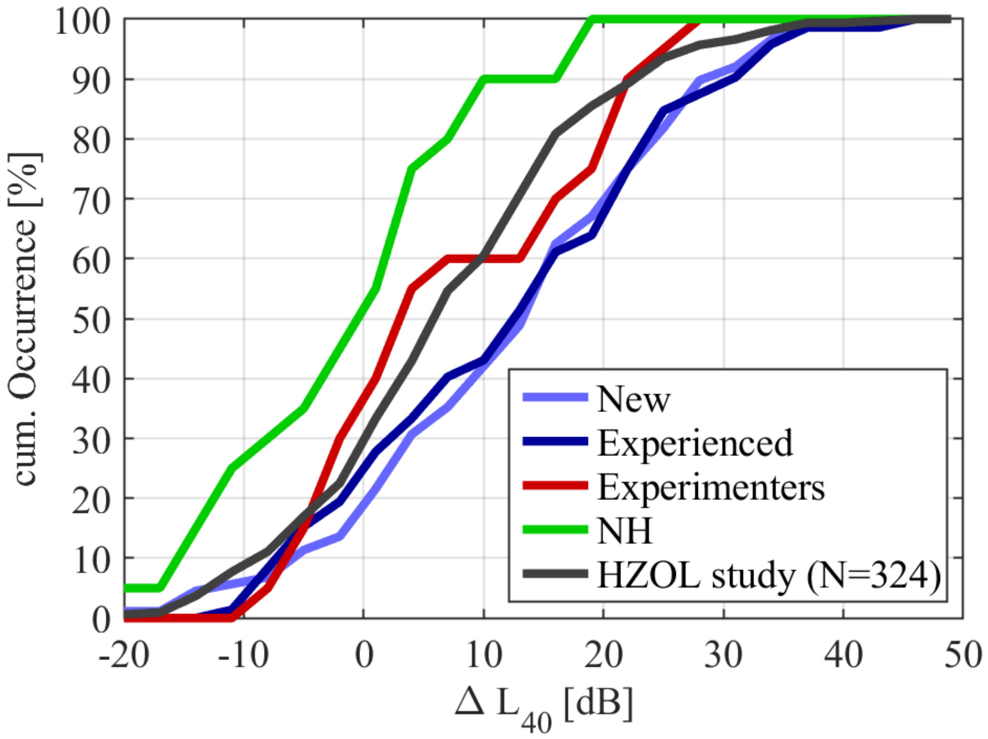

Figure S1 above shows the same information as Figure 4 from the main manuscript, but additionally includes the cumulative distribution of ΔL_{40} for a dataset of 324 hearing-impaired participants measured at the Hörzentrum Oldenburg (HZOL) during the development of the trueLOUDNESS procedure. All participants were part of a database of hearing-impaired individuals maintained by the HZOL, which were regularly invited to participate in hearing experiments. The distribution is in good agreement with the Experimenters group in the present dataset, which comprised 20 subjects from a similar database maintained by the German Institute of Hearing Aids, Lübeck.
